# Supplementary material for: SGLT2 Inhibitors in COVID-19: Umbrella Review, Meta-Analysis, and Bayesian Sensitivity Assessment
Source: Diseases. 2025 Feb 21;13(3):67. doi: 10.3390/diseases13030067 (PMC11941288; doi:10.3390/diseases13030067)
Supplement: Supplementary file 1 [file diseases-13-00067-s001.zip › Sup 6.pdf]

The adjusted search terms as per searched electronic databases [as of 5.12.2023]

Database	No	Search Query	Results
**NLM Clinical Trials.gov**			
	#1	("Sodium Glucose Transporter 2 Inhibitors" OR "SGLT-2 Inhibitors" OR "SGLT 2 Inhibitors" OR "SGLT2 Inhibitors" OR "Sodium-Glucose Transporter 2 Inhibitor" OR "Sodium Glucose Transporter 2 Inhibitor" OR "SGLT2 Inhibitor" OR "Inhibitor, SGLT2" OR "Gliflozins" OR "Gliflozin" OR "SGLT-2 Inhibitor" OR "Inhibitor, SGLT-2" OR "SGLT 2 Inhibitor" OR "canagliflozin" OR "dapagliflozin" OR "empagliflozin" OR "ertugliflozin" OR "ipragliflozin" OR "licogliflozin" OR "remogliflozin" OR "sergliflozin" OR "sotagliflozin" OR "tofogliflozin" OR "luseogliflozin" OR "bexagliflozin") AND (covid-19 OR covid)	2
**Cochrane**			
	#1	[mh "sars-cov-2"] OR "sars-cov-2" OR "covid" OR "covid-19" OR "covid-19"	
	#2	(Sodium Glucose Transporter 2 Inhibitors) OR (SGLT-2 Inhibitors) OR (SGLT 2 Inhibitors) OR (SGLT2 Inhibitors) OR (Sodium-Glucose Transporter 2 Inhibitor) OR (Sodium Glucose Transporter 2 Inhibitor) OR (SGLT2 Inhibitor) OR (Gliflozins) OR (Gliflozin) OR (SGLT-2 Inhibitor) OR (SGLT 2 Inhibitor) OR "canagliflozin" OR "dapagliflozin" OR "empagliflozin" OR "ertugliflozin" OR "ipragliflozin" OR "licogliflozin" OR "remogliflozin" OR "sergliflozin" OR "sotagliflozin" OR "tofogliflozin" OR "luseogliflozin" OR “bexagliflozin”	
	#3	#1 AND #2	24
**Embase**			
	#1	sars-cov-2'/exp OR 'sars-cov-2' OR 'covid'/exp OR 'covid' OR 'covid-19'/exp OR 'covid-19'	

#2	#1 OR 'coronavirus disease 2019'/dm
#3	sodium AND glucose AND transporter AND 2 AND inhibitors OR ('sglt 2' AND inhibitors) OR (sglt AND 2 AND inhibitors) OR (sglt2 AND inhibitors) OR ('sodium glucose' AND transporter AND 2 AND inhibitor) OR (sodium AND glucose AND transporter AND 2 AND inhibitor) OR (sglt2 AND inhibitor) OR gliflozins OR gliflozin OR ('sglt 2' AND inhibitor) OR (sglt AND 2 AND inhibitor) OR 'canagliflozin' OR 'dapagliflozin' OR 'empagliflozin' OR 'ertugliflozin' OR 'ipragliflozin' OR 'licogliflozin' OR 'remogliflozin' OR 'sergliflozin' OR 'sotagliflozin' OR 'tofogliflozin' OR 'luseogliflozin' OR 'bexagliflozin'
#4	('clinical article'/de OR 'clinical trial'/de OR 27,360,387) AND 5 AND dec AND 2023 AND 'clinical trial topic'/de OR 'human'/de OR 'major clinical study'/de OR 'multicenter study'/de OR 'observational study'/de OR 'phase 3 clinical trial topic'/de OR 'prospective study'/de OR 'randomized controlled trial'/de OR 'randomized controlled trial topic'/de
#5	'clinical article'/de OR 'clinical trial'/de OR 'clinical trial topic'/de OR 'human'/de OR 'major clinical study'/de OR 'multicenter study'/de OR 'observational study'/de OR 'phase 3 clinical trial topic'/de OR 'prospective study'/de OR 'randomized controlled trial'/de OR 'randomized controlled trial topic'/de
#6	'randomized controlled trial'/exp
#7	'controlled clinical trial'/de
#8	random*:ti,ab,tt

#9	'randomization'/de
#10	'intermethod comparison'/de
#11	placebo:ti,ab,tt
#12	compare:ti,tt OR compared:ti,tt OR comparison:ti,tt
#13	(evaluated:ab OR evaluate:ab OR evaluating:ab OR assessed:ab OR assess:ab) AND (compare:ab OR compared:ab OR comparing:ab OR comparison:ab)
#14	(open NEXT/1 label):ti,ab,tt
#15	((double OR single OR doubly OR singly) NEXT/1 (blind OR blinded OR blindly)):ti,ab,tt
#16	'double blind procedure'/de

#17	(parallel NEXT/1 group*):ti,ab,tt
#18	crossover:ti,ab,tt OR 'cross over':ti,ab,tt
#19	((assign* OR match OR matched OR allocation) NEAR/6 (alternate OR group OR groups OR intervention OR interventions OR patient OR patients OR subject OR subjects OR participant OR participants)):ti,ab,tt
#20	assigned:ti,ab,tt OR allocated:ti,ab,tt
#21	(controlled NEAR/8 (study OR design OR trial)):ti,ab,tt
#22	volunteer:ti,ab,tt OR volunteers:ti,ab,tt
#23	'human experiment'/de
#24	trial:ti,tt

#25	#6 OR #7 OR #8 OR #9 OR #10 OR #11 OR #12 OR #13 OR #14 OR #15 OR #16 OR #17 OR #18 OR #19 OR #20 OR #21 OR #22 OR #23 OR #24
#26	((random* NEXT/1 sampl* NEAR/8 ('cross section*' OR questionnaire* OR survey OR surveys OR database OR databases)):ti,ab,tt) NOT ('comparative study'/de OR 'controlled study'/de OR 'randomised controlled':ti,ab,tt OR 'randomized controlled':ti,ab,tt OR 'randomly assigned':ti,ab,tt)
#27	'cross-sectional study' NOT ('randomized controlled trial'/exp OR 'controlled clinical trial'/de OR 'controlled study'/de OR 'randomised controlled':ti,ab,tt OR 'randomized controlled':ti,ab,tt OR 'control group':ti,ab,tt OR 'control groups':ti,ab,tt)
#28	'case control*':ti,ab,tt AND random*:ti,ab,tt NOT ('randomised controlled':ti,ab,tt OR 'randomized controlled':ti,ab,tt)
#29	'systematic review':ti,tt NOT (trial:ti,tt OR study:ti,tt)
#30	nonrandom*:ti,ab,tt NOT random*:ti,ab,tt
#31	'random field*':ti,ab,tt
#32	('random cluster' NEAR/4 sampl*):ti,ab,tt

#33	review:ab AND review:it NOT trial:ti,tt
#34	'we searched':ab AND (review:ti,tt OR review:it)
#35	'update review':ab
#36	(databases NEAR/5 searched):ab
#37	(rat:ti,tt OR rats:ti,tt OR mouse:ti,tt OR mice:ti,tt OR swine:ti,tt OR porcine:ti,tt OR murine:ti,tt OR sheep:ti,tt OR lambs:ti,tt OR pigs:ti,tt OR piglets:ti,tt OR rabbit:ti,tt OR rabbits:ti,tt OR cat:ti,tt OR cats:ti,tt OR dog:ti,tt OR dogs:ti,tt OR cattle:ti,tt OR bovine:ti,tt OR monkey:ti,tt OR monkeys:ti,tt OR trout:ti,tt OR marmoset*:ti,tt) AND 'animal experiment'/de
#38	'animal experiment'/de NOT ('human experiment'/de OR 'human'/de)
#39	#26 OR #27 OR #28 OR #29 OR #30 OR #31 OR #32 OR #33 OR #34 OR #35 OR #36 OR #37 OR #38
#40	#25 NOT #39

	#41	#40 OR #4	
	#42	#2 AND #3	
	#43	#42 AND #41	
	#44	'review' OR 'meta analysis' OR 'meta-analysis'	
	#45	#41 OR #44	
	#46	#45 AND #42	448
**PubMed**			
	#1	((LitCTREATMENT[filter]) AND (LitCMECHANISM[filter])) OR COVID	
	#2	(Sodium Glucose Transporter 2 Inhibitors) OR (SGLT-2 Inhibitors) OR (SGLT 2 Inhibitors) OR (SGLT2 Inhibitors) OR (Sodium-Glucose Transporter 2 Inhibitor) OR (Sodium Glucose Transporter 2 Inhibitor) OR (SGLT2 Inhibitor]) OR (Inhibitor, SGLT2) OR (Gliflozins) OR (Gliflozin) OR (SGLT-2 Inhibitor) OR (Inhibitor, SGLT-2) OR (SGLT 2 Inhibitor) OR "canagliflozin" OR "dapagliflozin" OR "empagliflozin" OR "ertugliflozin" OR "ipragliflozin" OR "licogliflozin" OR	

	"remogliflozin" OR "sergliflozin" OR "sotagliflozin"OR "tofogliflozin" OR "luseogliflozin" OR "bexagliflozin")
#3	"cohort studies"[mesh] OR "case-control studies"[mesh] OR "comparative study"[pt] OR "risk factors"[mesh] OR "cohort"[tw] OR "compared"[tw] OR "groups"[tw] OR "case control"[tw] OR "multivariate"[tw]
#4	((systematic* [ti] AND review [ti]) OR Systematic overview* [ti] OR Cochrane review* [ti] OR systemic review* [ti] OR scoping review [ti] OR scoping literature review [ti] OR mapping review [ti] OR Umbrella review* [ti] OR (review of reviews [ti] OR overview of reviews [ti]) OR meta-review [ti] OR (integrative review [ti] OR integrated review [ti] OR integrative overview [ti] OR meta-synthesis [ti] OR metasynthesis [ti] OR quantitative review [ti] OR quantitative synthesis [ti] OR research synthesis [ti] OR meta-ethnography [ti]) OR Systematic literature search [ti] OR Systematic literature research [ti] OR meta-analyses [ti] OR metaanalyses [ti] OR metaanalysis [ti] OR meta-analysis [ti] OR meta-analytic review [ti] OR meta-analytical review [ti] OR meta-analysis [pt] OR ((search* [tiab] OR medline [tiab] OR pubmed [tiab] OR embase [tiab] OR Cochrane [tiab] OR scopus [tiab] or web of science [tiab] OR sources of information [tiab] OR data sources [tiab] OR following databases [tiab]) AND (study selection [tiab] OR selection criteria [tiab] OR eligibility criteria [tiab] OR inclusion criteria [tiab] OR exclusion criteria [tiab])) OR systematic review [pt]) NOT (letter [pt] OR editorial [pt] OR comment [pt] OR case reports [pt] OR historical article [pt] OR report [ti] OR protocol [ti] OR protocols [ti] OR withdrawn [ti] OR retraction of publication [pt] OR retraction of publication as topic [mesh] OR retracted publication [pt] OR reply [ti] OR published erratum [pt])
#5	randomized controlled trial [pt]
#6	controlled clinical trial [pt]
#7	randomized [tiab]
#8	placebo [tiab]
#9	clinical trials as topic [mesh: noexp]

#10	randomly [tiab]	
#11	trial [ti]	
#12	#5 OR #6 OR #7 OR #8 OR #9 OR #10 OR #11	
#13	animals [mh] NOT humans [mh]	
#14	#12 NOT #13	
#15	#1 AND #2 AND (#3 OR #4 OR #14)	85
