# Supplementary material for: SGLT2 Inhibitors in COVID-19: Umbrella Review, Meta-Analysis, and Bayesian Sensitivity Assessment
Source: Diseases. 2025 Feb 21;13(3):67. doi: 10.3390/diseases13030067 (PMC11941288; doi:10.3390/diseases13030067)
Supplement: Supplementary file 1 [file diseases-13-00067-s001.zip › Supp Table 1.pdf]

Supplementary Table 1. The PICO (Population, Intervention, Comparator, Outcomes) elements of the research questions of the review

	Research question 1	Research question 2	Research question 3
**Population**	Hospitalized COVID-19 patients	COVID-19 patients	COVID-19 patients
**Intervention**	SGLT2i	History of SGLT2i use	History of SGLT2i use
**Comparator**	Placebo	No history of SGLT2i use	No history of SGLT2i use
**Outcomes**	<u>Efficacy:</u><br>Mortality, need for mechanical ventilation, ICU admission, hospital discharge, and other clinically relevant and statistically homogenous outcomes at each time point reported in 2 or more studies<br><br><u>Safety:</u><br>Diabetic ketoacidosis, acute kidney injury, and other clinically relevant and statistically homogenous outcomes at each time point reported in 2 or more studies	<u>Efficacy:</u><br>Mortality, need for mechanical ventilation, ICU admission, hospitalization, and other clinically relevant and statistically homogenous outcomes reported in 2 or more studies<br><br><u>Safety:</u><br>Diabetic ketoacidosis, acute kidney injury, and other clinically relevant and statistically homogenous outcomes reported in 2 or more studies	<u>Efficacy:</u><br>Mortality, need for mechanical ventilation, ICU admission, hospitalization, and other clinically relevant and statistically homogenous outcomes reported in 2 or more studies<br><br><u>Safety:</u><br>Diabetic ketoacidosis, acute kidney injury, and other clinically relevant and statistically homogenous outcomes reported in 2 or more studies
**Study design**	Randomized controlled trials	All original studies (interventional or observational) answering the above research question	All systematic reviews and meta-analysis
**Statistical measure**	Risk ratio	Odds ratio	Odds ratio
