# Supplementary material for: SGLT2 Inhibitors in COVID-19: Umbrella Review, Meta-Analysis, and Bayesian Sensitivity Assessment
Source: Diseases. 2025 Feb 21;13(3):67. doi: 10.3390/diseases13030067 (PMC11941288; doi:10.3390/diseases13030067)
Supplement: Supplementary file 1 [file diseases-13-00067-s001.zip › Supp table 2.pdf]

Supplementary table 2. GRADE Assessment of SGLT2 Inhibitor Effects on COVID-19 Outcomes

Certainty assessment							№ of patients		Effect	Certainty	Importance
No of studies	Study design	Risk of bias	Inconsistency	Indirectness	Imprecision	Other considerations	SGLT2i use	Non-use of SGLT2i	Odds Ratio (95% CI)		
**Mortality**											
12	Non-randomised studies	Not serious	Not serious	Not serious	Not serious	None	20418	104254	OR 0.67 (0.53 to 0.84)	⊕⊕◯◯ Low	CRITICAL
**Hospitalization**											
8	Non-randomised studies	Not serious	Not serious	Not serious	Not serious	None	26827	165230	OR 0.84 (0.75 to 0.94)	⊕⊕◯◯ Low	CRITICAL
**ICU admission**											
3	Non-randomised studies	Not serious	Serious[a]	Not serious	Very serious[b]	None	5801	60046	OR 0.93 (0.82 to 1.06)	⊕◯◯◯ Very low	CRITICAL
**Mechanical ventilation**											
3	Non-randomised studies	Not serious	Not serious	Not serious	Very serious[b]	None	3763	10374	OR 0.82 (0.50 to 1.36)	⊕◯◯◯ Very low	CRITICAL
**Emergency visits**											
1	Non-randomised studies	Not serious	Not serious	Not serious	Very serious[b]	None	3504	9650	OR 0.96 (0.88 to 1.04)	⊕◯◯◯ Very low	CRITICAL
**Diabetic ketoacidosis**											
2	Non-randomised studies	Not serious	Not serious	Not serious	Very serious[c]	None	283	3029	OR 1.20 (0.64 to 2.27)	⊕◯◯◯ Very low	CRITICAL
**Acute kidney injury**											
1	Non-randomised studies	Not serious	Not serious	Not serious	Very serious[b]	None	53	192	OR 0.81 (0.44 to 1.49)	⊕◯◯◯ Very low	CRITICAL

CI: confidence interval; **OR:** odds ratio

Explanations

a. The point estimates point in both directions. Thus, the evidence certainty is downgraded by one level for inconsistency.
b. The point estimate suggests benefit, and the CI includes the possibility of important harm. Thus, the evidence certainty is downgraded by two levels for imprecision.
c. The point estimate suggests harm, and the CI includes the possibility of important benefit. Thus, the evidence certainty is downgraded by two levels for imprecision.
