# Supplementary material for: SGLT2 Inhibitors in COVID-19: Umbrella Review, Meta-Analysis, and Bayesian Sensitivity Assessment
Source: Diseases. 2025 Feb 21;13(3):67. doi: 10.3390/diseases13030067 (PMC11941288; doi:10.3390/diseases13030067)
Supplement: Supplementary file 1 [file diseases-13-00067-s001.zip › Supp Table S3.pdf]

Supplementary table 3. Critical Appraisal for observational studies using the ROBINS-I tool for non-randomized studies of interventions

Author - Year	Confounding	Selection of participants	Classification of interventions	Deviations from intended interventions	Missing data	Measurement of outcomes	Selection of reported results	Overall
Kahkoska 2021	Serious	Moderate	Moderate	Low	Low	Moderate	Low	Serious
Israelsen 2021	Serious	Moderate	Moderate	Low	Low	Moderate	Low	Serious
Monda 2023	Serious	Moderate	Moderate	Low	Low	Low	Low	Serious
Ozbek 2023	Serious	Critical	Moderate	Low	Low	Moderate	Low	Critical
Salgado-Barreira 2023	Moderate	Low	Moderate	Low	Low	Moderate	Low	Moderate
Foresta 2023	Serious	Moderate	Moderate	Low	Low	Moderate	Low	Serious
Khunti 2022	Serious	Moderate	Moderate	Low	Low	Moderate	Low	Serious
Israel 2021	Moderate	Moderate	Moderate	Low	Low	Low	Low	Moderate
Min 2022	Moderate	Low	Moderate	Low	Low	Low	Low	Moderate
Ramos-Rincón 2021	Serious	Moderate	Moderate	Low	Serious	Moderate	Low	Serious
Orioli 2020	Serious	Moderate	Moderate	Low	Serious	Moderate	Low	Serious
Wander 2021	Serious	Moderate	Moderate	Low	Low	Low	Low	Serious
Shestakova 2022	Serious	Moderate	Moderate	Low	No information	Moderate	Low	Serious

Dalan 2021	Serious	Moderate	Moderate	Low	No information	Moderate	Moderate	Serious
Silverii 2020	Serious	Moderate	Moderate	Low	No information	Low	Low	Serious
Yeh 2022	Serious	Moderate	Moderate	Low	Low	Low	Low	Serious
Sourij 2020	Serious	Moderate	Moderate	Low	Low	Moderate	Low	Serious
