# Supplementary material for: SGLT2 Inhibitors in COVID-19: Umbrella Review, Meta-Analysis, and Bayesian Sensitivity Assessment
Source: Diseases. 2025 Feb 21;13(3):67. doi: 10.3390/diseases13030067 (PMC11941288; doi:10.3390/diseases13030067)
Supplement: Supplementary file 1 [file diseases-13-00067-s001.zip › Table S4.pdf]

Supplementary table 4. Critical Appraisal Tool for the Risk of Bias in Systematic Reviews from the Joanna Briggs Institute for Systematic Reviews and Research Syntheses

Title	Author	Year	1. PICO components	2. Review methods	3. Study selection	4. Literature search	5. Study selection	6. Data extraction	7. Exclusion justification	8. Study description	9. Included studies risk of bias	10. Included studies funding	11. Statistical methods	12. Risk of bias impact	13. Risk of bias accountability	14. Heterogeneity	15. Publication bias	16. Conflict of interest	Results
Association of Glucose-Lowering Drugs With Outcomes in Patients With Diabetes Before Hospitalization for COVID-19:	Zhu et.al.	2022	Y	PY	Y	Y	Y	Y	N	PY	Y	N	Y	Y	Y	N	Y	Y	Included

A Systematic Review and Network Meta-analysis.																			
The Association Between Antidiabetic Agents and Clinical Outcomes of COVID-19 Patients With Diabetes: A Bayesian Network Meta-Analysis.	Chen et.al.	2022	Y	Y	Y	Y	N	Y	N	PY	Y	Y	Y	Y	Y	Y	Y	Y	Included
Sodium-glucose cotransporter-2 inhibitor-	Khedr st.al.	2023	Y	Y	Y	Y	Y	Y	PY	PY	N	N	Y	N	N	Y	Y	Y	Included

associated euglycemic diabetic ketoacidosis in COVID-19-infected patients: A systematic review of case reports																			
Effect of Antidiabetic Therapy on Clinical Outcomes of COVID-19 Patients With Type 2 Diabetes: A Systematic Review and Meta-Analysis	Zhan	2023	Y	PY	Y	Y	Y	Y	N	PY	Y	Y	Y	Y	Y	Y	Y	Y	Included
Noninsulin	Nassar	2023	Y	N	Y	Y	Y	Y	PY	Y	Y	Y	Y	Y	Y	Y	Y	Y	Included

-based antihyperglycemic medications in patients with diabetes and COVID-19: A systematic review and meta-analysis																			
Pre-admission use of sodium glucose transporter-2 inhibitor (SGLT-2i) may significantly improves Covid-19 outcomes in patients with diabetes:	Permana	2023	Y	Y	Y	Y	Y	Y	PY	PY	Y	Y	Y	Y	Y	Y	Y	Y	Included

A systematic review, meta-analysis, and meta-regression																			
Effects of novel glucose-lowering drugs on the COVID-19 patients with diabetes: A network meta-analysis of clinical outcomes	Yang	2023	Y	Y	Y	Y	Y	Y	N	PY	Y	Y	Y	Y	Y	Y	Y	Y	Included
Preadmission use of antidiabetic medications and mortality among	Nguyen	2022	Y	Y	Y	Y	Y	Y	N	PY	Y	Y	Y	Y	Y	Y	Y	Y	Included

patients with COVID-19 having type 2 diabetes: A meta-analysis																			
Association Between Anti-diabetic Agents and Clinical Outcomes of COVID-19 in Patients with Diabetes: A Systematic Review and Meta-Analysis	Han	2022	Y	Y	Y	Y	Y	Y	N	PY	Y	Y	Y	N	N	Y	Y	Y	Included
Diabetes-related acute metabolic	Papadopoulos	2021	Y	Y	Y	Y	Y	Y	N	Y	Y	Y	Y	Y	Y	Y	Y	Y	Included

emergencies in COVID-19 patients: a systematic review and meta-analysis																		
