# Supplementary figures and images for: SGLT2 Inhibitors in COVID-19: Umbrella Review, Meta-Analysis, and Bayesian Sensitivity Assessment
Source: Diseases. 2025 Feb 21;13(3):67. doi: 10.3390/diseases13030067 (PMC11941288; doi:10.3390/diseases13030067)

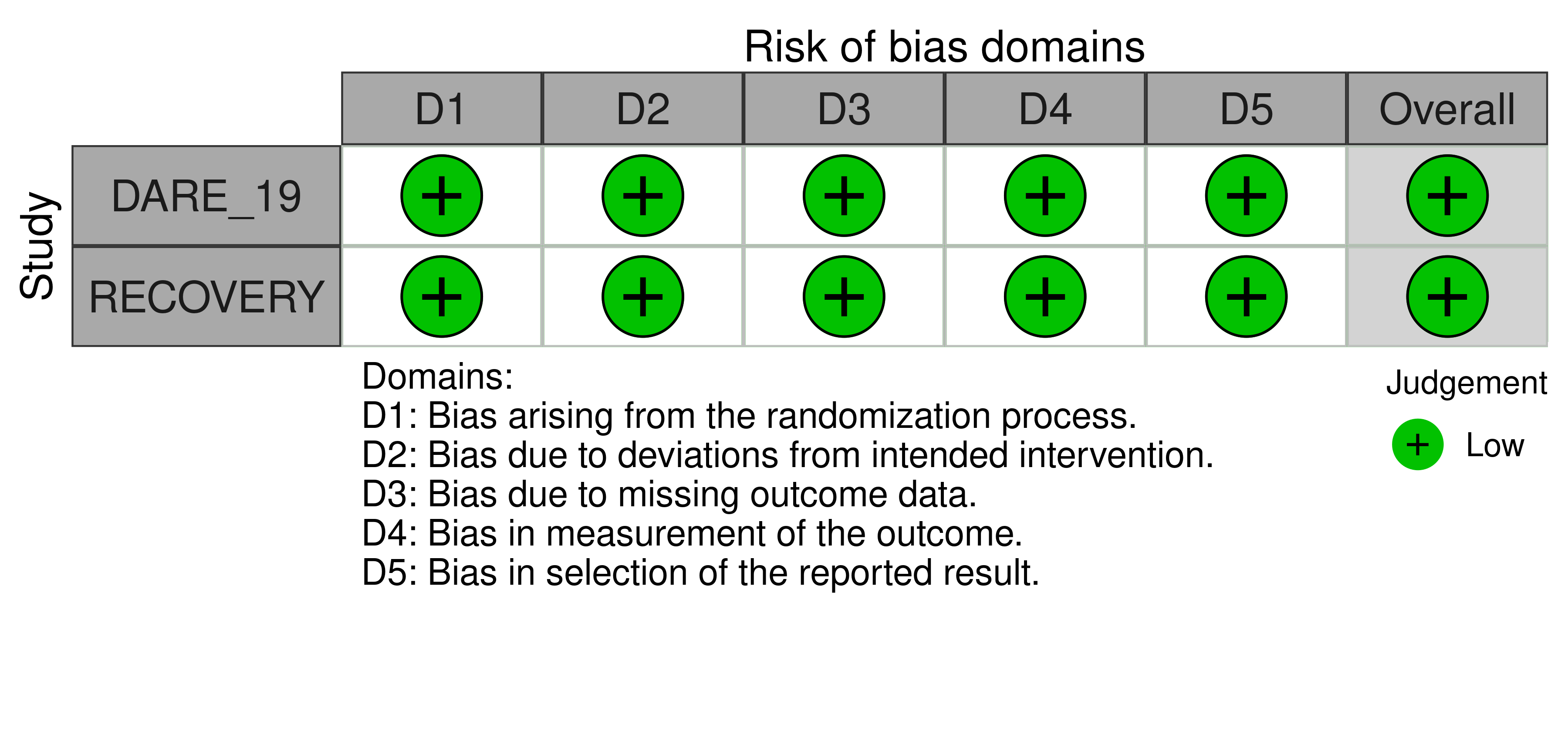

Supplement: Supplementary file 1 [file diseases-13-00067-s001.zip › Supp f1.png]

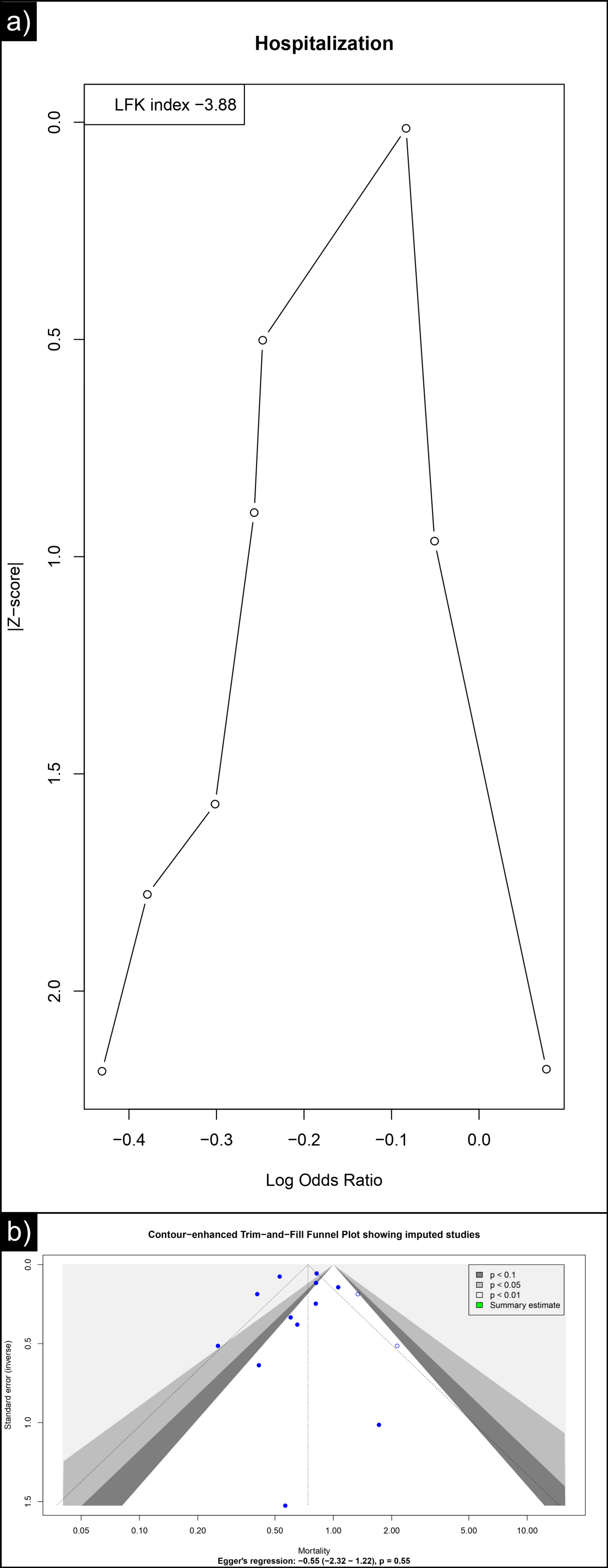

Supplement: Supplementary file 1 [file diseases-13-00067-s001.zip › Supp fig 2.png]

a)

Hospitalization

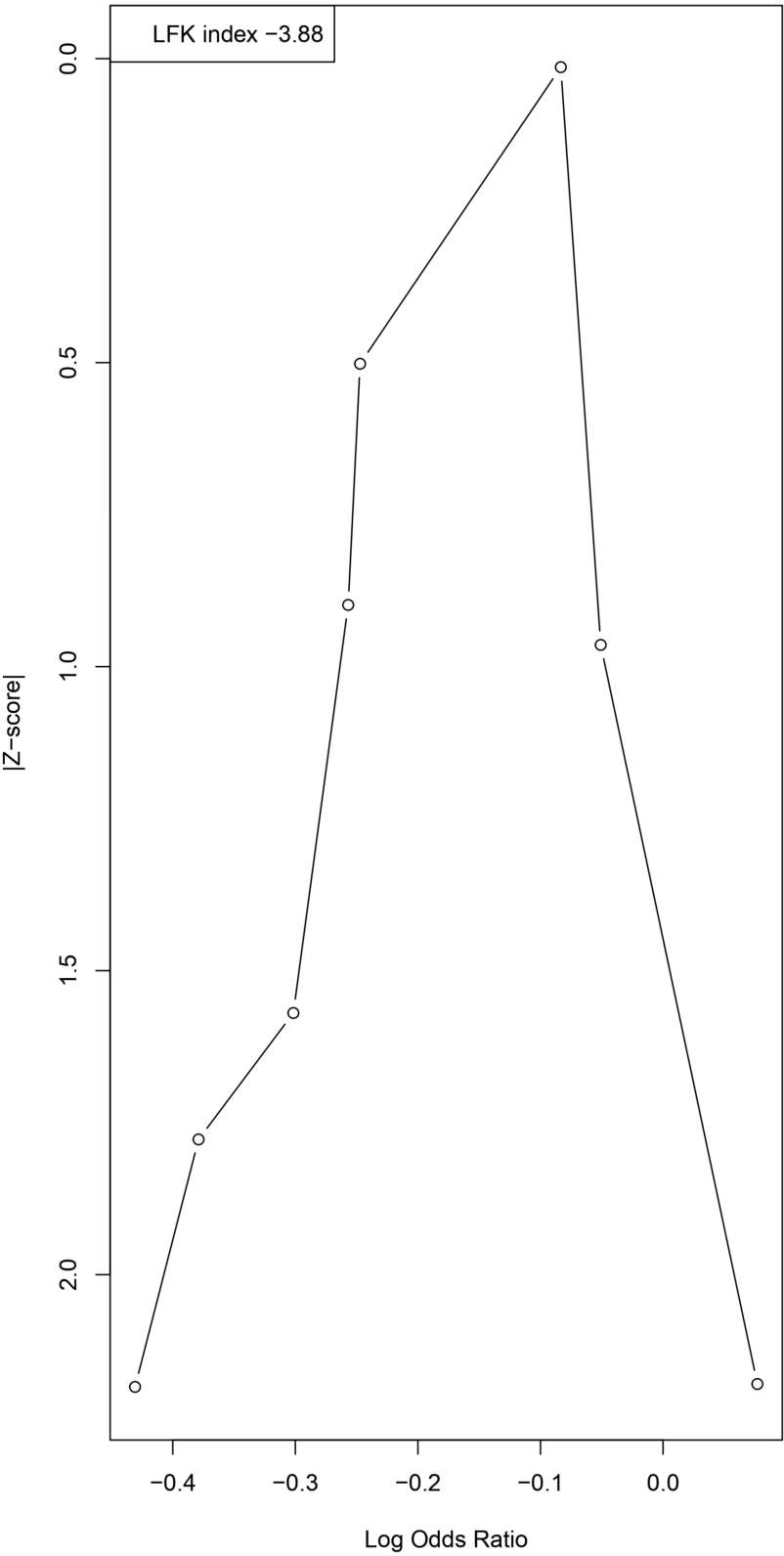

b)

Contour-enhanced Trim-and-Fill Funnel Plot showing imputed studies

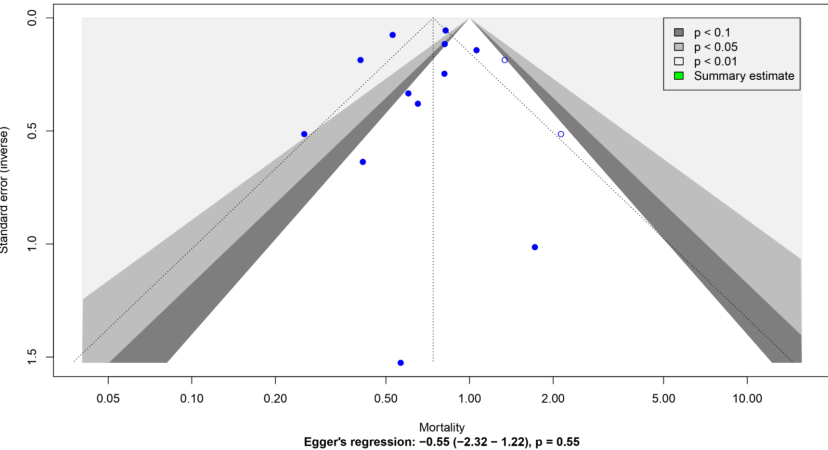

Supplement: Supplementary file 1 [file diseases-13-00067-s001.zip › supple f2.pdf]
